# Supplementary material for: Impact of early pregnancy body mass index and gestational weight gain on birth outcomes: Findings from a pregnancy cohort in South Delhi, India
Source: PLOS Glob Public Health. 2026 Feb 9;6(2):e0005932. doi: 10.1371/journal.pgph.0005932 (PMC12885321; doi:10.1371/journal.pgph.0005932)
Supplement: S1 Table — Association between early pregnancy body mass index and gestational weight gain with infant weight across pregnancy. Values were calculated using GLMs of the Gaussian family with an identity-link function, compared with reference value, normal BMI with AGWG, and presented as β-coefficient and 95% CIs. AGWG, adequate gestational weight gain; BMI, body mass index; CI, confidence interval; EGWG, excessive gestational weight gain; GLM, generalized linear model; IGWG, inadequate gestational weight gain. 1Adjusted variables: maternal age, maternal education, family wealth quintile and intervention. (DOCX) [file pgph.0005932.s001.docx]

**S1 Table**.

|  | **Weight, g: β coefficient (95% CI)** | | | |
| --- | --- | --- | --- | --- |
| **Variables** | **Unadjusted** | ***P* value** | **Adjusted^1^** | ***P* value** |
| ***Total GWG (from enrolment to last visit of before delivery) (n=3239)*** | | | | |
| BMI 18·5 – <25·0 and AGWG | Ref |  | Ref |  |
| BMI 18·5 – <25·0 and IGWG | -124·78(-161.90‒ -87·66) | 0.000 | -126.62(-163.78‒ -89.46) | 0.000 |
| BMI 18·5 – <25·0 and EGWG | 89·16(37·29‒141·03) | 0.001 | 92.22(40.39‒144.04) | 0.000 |
| BMI<18·5 and AGWG | -86·16(-162·26‒ -10·06) | 0.026 | -75.35(-151.49‒0.80) | 0.052 |
| BMI<18·5 and IGWG | -246·24(-296·39‒ -196·09) | 0.000 | -239.12(-289.53‒ -188.72) | 0.000 |
| BMI<18·5 and EGWG | 45·10(-77·63‒167·82) | 0.471 | 54.63(-67.87‒177.13) | 0.382 |
| BMI≥25·0 and AGWG | 26.73·42(-33·18‒86·64) | 0.382 | 14.28(-46.10‒74.66) | 0.643 |
| BMI≥25·0 and IGWG | -2·14(-53.97‒49·68) | 0.935 | -11.28(-63.47‒40.92) | 0.672 |
| BMI≥25·0 and EGWG | 128·52(77·97‒179·07) | 0.000 | 116.26(65.38‒167.14) | 0.000 |
| ***GWG from enrolment to 26 weeks of gestation (n=3502)*** | | | | |
| BMI 18·5 – <25·0 and AGWG | Ref |  | Ref |  |
| BMI 18·5 – <25·0 and IGWG | -90·41(-130·29‒ -50·52) | 0.000 | -87.08(-127.26‒ -46.90) | 0.000 |
| BMI 18·5 – <25·0 and EGWG | 115.37(59·35‒171·39) | 0.000 | 114.22(58.24‒170.20) | 0.000 |
| BMI<18·5 and AGWG | -83·00(-159·31‒ -6·69) | 0.033 | -78.2(-154.66‒ -1.74) | 0.045 |
| BMI<18·5 and IGWG | -247·60(-302·86‒ -192·32) | 0.000 | -241.74(-297.34‒ -186.14) | 0.000 |
| BMI<18·5 and EGWG | -25·30(-143·22‒92.63) | 0.674 | -18.18(-136.04‒99.68) | 0.762 |
| BMI≥25·0 and AGWG | 127·12(60·26‒193·98) | 0.000 | 122.08(54.75‒189.42) | 0.000 |
| BMI≥25·0 and IGWG | -15·20(-67·28‒36·89) | 0.567 | -13.40(-66.13‒39.33) | 0.618 |
| BMI≥25·0 and EGWG | 116·44(58·97‒173·91) | 0.000 | 109.54(51.52‒167.57) | 0.000 |
| ***GWG from 27 weeks to last visit of before delivery (n=2949)*** | | | | |
| BMI 18·5 – <25·0 and AGWG | Ref |  | Ref |  |
| BMI 18·5 – <25·0 and IGWG | -91·40(-134·29‒ -48·51) | 0.000 | -91.61(-134.57‒ -48.64) | 0.000 |
| BMI 18·5 – <25·0 and EGWG | 34·44(-12·11‒80·98) | 0.147 | 34.63(-11.99‒81.24) | 0.145 |
| BMI<18·5 and AGWG | -102·22(-189·73, -14·70) | 0.022 | -96.86(-184.43‒ -9.30) | 0.030 |
| BMI<18·5 and IGWG | -230·29(-286·81‒ -173·76) | 0.000 | -223.23(-280.12‒ -166.35) | 0.000 |
| BMI<18·5 and EGWG | -42·07(-150·07‒65·92) | 0.445 | -32.98(-141.12‒75.16) | 0.550 |
| BMI≥25·0 and AGWG | 60·11(-12·47‒132·69) | 0.104 | 53.18(-19.61‒125.97) | 0.152 |
| BMI≥25·0 and IGWG | 66·84(-2·33‒136·01) | 0.058 | 59.40(-10.15‒128.95) | 0.094 |
| BMI≥25·0 and EGWG | 92·38(43·33‒141·42) | 0.000 | 84.51(35.08‒133.95) | 0.001 |
