# Supplementary material for: Impact of early pregnancy body mass index and gestational weight gain on birth outcomes: Findings from a pregnancy cohort in South Delhi, India
Source: PLOS Glob Public Health. 2026 Feb 9;6(2):e0005932. doi: 10.1371/journal.pgph.0005932 (PMC12885321; doi:10.1371/journal.pgph.0005932)
Supplement: S2 Table — Values were calculated using GLMs of the Gaussian family with an identity-link function, compared with reference value, normal BMI with AGWG, and presented as β-coefficient and 95% CIs. AGWG, adequate gestational weight gain; BMI, body mass index; CI, confidence interval; EGWG, excessive gestational weight gain; GLM, generalized linear model; IGWG, inadequate gestational weight gain.1Adjusted variables: maternal age, maternal education, family wealth quintile and intervention. (DOCX) [file pgph.0005932.s002.docx]

**S2 Table**

|  | **Length, cm: β coefficient (95% CI)** | | | |
| --- | --- | --- | --- | --- |
| **Variables** | **Unadjusted** | ***P* value** | **Adjusted^1^** | ***P* value** |
| ***Total GWG (from enrolment to last visit of before delivery) (n=3239)*** | | | | |
| BMI 18·5 – <25·0 and AGWG | Ref |  | Ref |  |
| BMI 18·5 – <25·0 and IGWG | -0·42(-0·61‒ -0·24) | 0.000 | -0.47(-0.65‒ -0.28) | 0.000 |
| BMI 18·5 – <25·0 and EGWG | 0·22(-0·03‒0·48) | 0.090 | 0.26(0.01‒0.52) | 0.047 |
| BMI<18·5 and AGWG | -0·36(-0·73‒0·02) | 0.064 | -0.31(-0.68‒0.07) | 0.111 |
| BMI<18·5 and IGWG | -0·99(-1·24‒ -0·74) | 0.000 | -0.97(-1.22‒ -0.72) | 0.000 |
| BMI<18·5 and EGWG | 0·26(-0·35‒0·87) | 0.406 | 0.33(-0.28‒0.94) | 0.284 |
| BMI≥25·0 and AGWG | 0·01(-0·29‒0·30) | 0.967 | -0.07(-0.37‒0.23) | 0.635 |
| BMI≥25·0 and IGWG | 0·07(-0·19‒0·32) | 0.601 | -0.02(-0.28‒0.24) | 0.881 |
| BMI≥25·0 and EGWG | 0·32(0·07‒0·57) | 0.012 | 0.26(0.01‒0.51) | 0.046 |
| ***GWG from enrolment to 26 weeks of gestation (n=3502)*** | | | | |
| BMI 18·5 – <25·0 and AGWG | Ref |  | Ref |  |
| BMI 18·5 – <25·0 and IGWG | -0·37(-0·57‒ -0·17) | 0.000 | -0.39(-0.59‒ -0.19) | 0.000 |
| BMI 18·5 – <25·0 and EGWG | 0·41(0·13‒0·69) | 0.004 | 0.42(0.14‒0.70) | 0.003 |
| BMI<18·5 and AGWG | -0·33(-0·71‒0·06) | 0.094 | -0.32(-0.70‒0.07) | 0.107 |
| BMI<18·5 and IGWG | -1·14(-1·42‒ -0·86) | 0.000 | -1.12(-1.40‒ -0.84) | 0.000 |
| BMI<18·5 and EGWG | -0·18(-0·77‒0·41) | 0.551 | -0.15(-0.74‒0.44) | 0.624 |
| BMI≥25·0 and AGWG | 0·38(0·04‒0·71) | 0.027 | 0.33(-0.01‒0.67) | 0.054 |
| BMI≥25·0 and IGWG | -0·10(-0·36‒0·16) | 0.448 | -0.13(-0.40‒0.13) | 0.322 |
| BMI≥25·0 and EGWG | 0·21(-0·08‒0·50) | 0.147 | 0.17(-0.12‒0.46) | 0.248 |
| ***GWG from 27 weeks to last visit of before delivery (n=2949)*** | | | | |
| BMI 18·5 – <25·0 and AGWG | Ref |  | Ref |  |
| BMI 18·5 – <25·0 and IGWG | -0·24(-0·45‒ -0·03) | 0.023 | -0.26(-0.47‒ -0.05) | 0.015 |
| BMI 18·5 – <25·0 and EGWG | 0·04(-0·18‒0·27) | 0.707 | 0.06(-0.17‒0.29) | 0.617 |
| BMI<18·5 and AGWG | -0·42(-0·85‒0·01) | 0.056 | -0.39(-0.82‒0.03) | 0.071 |
| BMI<18·5 and IGWG | -0·93(-1·21‒ -0·65) | 0.000 | -0.91(-1.18‒ -0.63) | 0.000 |
| BMI<18·5 and EGWG | -0·22(-0·75‒0·31) | 0.416 | -0.15(-0.68‒0.37) | 0.567 |
| BMI≥25·0 and AGWG | 0·15(-0·20‒0·51) | 0.406 | 0.11(-0.25‒0.46) | 0.559 |
| BMI≥25·0 and IGWG | 0·34(-0·01‒0·68) | 0.049 | 0.28(-0.06‒0.62) | 0.110 |
| BMI≥25·0 and EGWG | 0·22(-0·02‒0·46) | 0.076 | 0.18(-0.07‒0.42) | 0.153 |
