# Supplementary material for: Impact of early pregnancy body mass index and gestational weight gain on birth outcomes: Findings from a pregnancy cohort in South Delhi, India
Source: PLOS Glob Public Health. 2026 Feb 9;6(2):e0005932. doi: 10.1371/journal.pgph.0005932 (PMC12885321; doi:10.1371/journal.pgph.0005932)
Supplement: Attachment — Submitted filename: Peer review of Article for PLOS.docx [file pgph.0005932.s008.docx]

**Peer Review for PLOS Global Public Health:**

By Dr.Chandrakala Maran

HOD, OBGYN Dept, K.G.Hospital And PG Institute, Coimbatore,INDIA

**Article:**

Impact of early pregnancy body mass index and gestational weight gain on birth

outcomes: Findings from a pregnancy cohort in South Delhi, India

**Introduction:**

The background and the aim that have been identified for the study is commendable and of social importance. Low birth weight has been identified as one of the causes of Metabolic syndrome in that Index child and prevention of Low birth weight by identifying the reason in the Antenatal period would reduce the incidence of individuals with metabolic syndromes in a society. Stunting and Wasting at birth also have long term implications and should be prevented.

**Materials and methods**:

The analysis used data from Women and Infants Integrated Intervention for Growth Study

(WINGS). 4 authors of the WINGS are involved in this study. Women recruited in the previous study (WINGS) - those with interventions and control group were followed up till they became pregnant. Those who became pregnant were recruited for this current study and randomised again to receive interventions or receive routine care.

The data collection method is clear. Methods of Classification of the Subjects based on BMI and follow up of the recruited subjects for Gestational weight gain has been explained adequately. The method used for the calculation of Gestational age at Birth needs more clarity.Measurements of Newborns which are needed to assess Stunting and Wasting has been mentioned clearly.

**Enrolment of the subjects in the current study:**

The process of enrolment has to be worded appropriately as follows for better understanding:

Women from the previous study who became pregnant within 18 months of enrolment were enrolled for this study.Their BMI was calculated for classification into Women who were Underweight, Normal, Overweight and Obese. They were randomised to receive intervention or routine care.

**Definition of primary exposure variables:**

Explained clearly.

**Definition of outcome variables:**

Details of measurements of Continuous and Categorical variables have been explained well and it is found that the authors have adhered to previous prescribed standards.

Calculation of Exact Gestational age at birth need clarity.

**Inclusion criteria:**

Should be described as Antenatal women with Gestational age <14 weeks

Singleton pregnancy.

Neonates delivered by these women within <14 days of birth.

Further there is no comment on inclusion or exclusion of women with comorbidities which could be confounding factors.

**Power Calculation and Statistical methods:**

Seem to be appropriate.

**Table1:**

Under this topic, the following lines may be removed, because Table 1 describes Sociodemographic profiles of Enrolled Subjects.

The overall prevalence of inadequate, adequate, and excessive GWG was 47·4%, 31·2%, and

238 21·4% respectively. Mean (SD) of newborns weight and length were 2729.9 (419.7) g and 239 48.1 (2.1) cm respectively

**Distribution of adverse birth outcomes by early pregnancy BMI and GWG:**

Under this Topic,the following sentence may be rephrased or removed.

The overall prevalence of term SGA, LBW, preterm and stunting are shown by GWG and

254 early pregnancy BMI

**Association between early pregnancy BMI, GWG, and preterm:**

Confounding factors which were considered for adjustment to be mentioned.

**Interaction between early pregnancy BMI and GWG:**

Figures mentioned as 4A and 4B are missing.

**Discussion:**

Appropriate.

Strengths and Limitations have been addressed well.

Among Limitations, the potential confounding factors not mentioned are Gestational diabetes,Hypertension ,Anemia and other comorbidities which could have influenced Gestational weight gain and Infant birth measurements.

**Conclusion :**

Well conceived and expressed.
