# Supplementary material for: Impact of early pregnancy body mass index and gestational weight gain on birth outcomes: Findings from a pregnancy cohort in South Delhi, India
Source: PLOS Glob Public Health. 2026 Feb 9;6(2):e0005932. doi: 10.1371/journal.pgph.0005932 (PMC12885321; doi:10.1371/journal.pgph.0005932)
Supplement: Attachment — Submitted filename: Response to reviewers_20.12.2025.docx [file pgph.0005932.s009.docx]

**REVIEWER’S COMMENTS**

PLOS Global Public Health

Impact of early pregnancy body mass index and gestational weight gain on birth outcomes: Findings from a pregnancy cohort in South Delhi, India.

Manuscript ID: PGPH-D-25-02926

**Comments of Reviewer-1**

**Title-OK**

Response: Thanks

**Abbreviations- Give the list of all abbreviations with their long term**

Response: List of all abbreviation has been added in manuscript (line 26-39).

“AGWG, Adequate Gestational Weight Gain; BMI, Body Mass Index; CI, Confidence Interval

EGWG, Excessive Gestational Weight Gain; GLM, Generalized Linear Model; GWG, Gestational Weight Gain; IGWG, Inadequate Gestational Weight Gain; IOM, Institute of medicine; LBW, Low Birth Weight; LMICs, Low- and Middle-Income Countries; RR, Risk Ratio; SGA, Small for Gestational Age; WINGS, Women and Infants Integrated Intervention for Growth Study”.

**Abstract- Write the name of corresponding author and email**

Response: Thank you. Name and email address of corresponding author have been added in the revised manuscript (line 14 and 15)

**Abstract- Write the section of abstract into structured paragraphs**

Response: Thank you for the observation. The Background (line 49), Objective (line 55), Methods (line 59), Results (line 65), and Conclusion (line 74) are presented as separate paragraphs in the revised manuscript.

**Abstract- The highest prevalence of SGA (62.4%) was observed among underweight women**

**with IGWG. Give the CI for this prevalence value don’t leave it alone**

Response: Prevalence of SGA with 95%CI (62.4%; 95%CI 57.1‒67.5) has been added in line 65 and throughout the manuscript.

**Introduction- OK**

Response: Thanks

**Research questions or hypothesis-OK**

Response: Thanks

**Methodology- Prepregnant women were enrolled from 1 July 2017 to 30 December 2019 and were prospectively followed, and birth outcomes were collected upto 10 July 2021 “State the follow up time by saying were followed for the period of …. Years from ….. to …..”**

Response: Thanks for the suggestion. We have made the suggested changes (line 148-150).

“Prepregnant women were enrolled from 1 July 2017 to 30 December 2019 and were followed for the period of 18 months, from 1 January 2020 to 30 June 2021”.

**Inclusion criteria- The following inclusion criteria were set for this analysis: <14 weeks of**

**197 gestational age at enrolment, singleton pregnancy and birth outcomes were measured <14**

**198 days of birth. OK**

Response: Thanks

**Exclusion criteria- Include the exclusion criteria in your work. What were the exclusion criterias?**

Response: Exclusion criteria has been added in the revised manuscript (line 226-227).

“Women living in temporary housing and those moving away from study area were excluded”.

**Analysis- All analyses were performed using Stata version 14 (StataCorp, College Station, TX, USA), with statistical significance defined as p < 0.05. “Include the words from 95% CI at the end of this sentence”**

Response: 95%CI has been added in the revised manuscript (line 257).

“Statistical significance was defined as *p* < 0.05, with 95%CI”

**Ethical issues- Show how the confidentiality, Privacy and consenting was done as well as how participants were enrolled ethically**

Response: ‘Ethics statement’ has been added as sub-heading under the main heading in the revised manuscript accordingly (line 135-143).

“The Ethics Review Committee of the Society for Applied Studies, New Delhi (SAS/ ERC/LG/2027), Vardhman Medical College, Safdarjung Hospital, New Delhi (IEC/SJH/VMMC/PROJECT-2017/694) and the World Health Organization, Geneva (ERC.0002934) have approved the study. The study was conducted in accordance with the ethical principles outlined in the Declaration of Helsinki. The trial was registered with the Clinical Trials Registry of India (CTRI/2017/06/008908). Written informed consent was obtained from all mothers prior to participation. All data were collected in a private area of the participants’ homes, and confidentiality of the collected data was maintained”.

**Results- The overall prevalence of inadequate, adequate, and excessive GWG was 47·4%, 31·2%, and 21·4% respectively. “Put the CI in this prevalence”**

Response: 95%CI has been added with all prevalence in the revised manuscript (line 271-273).

“The overall prevalence of inadequate, adequate, and excessive GWG was 47·4% (95%CI 45.8‒49.0), 31·2% (95%CI 29.8‒32.7), and 21·4% (20.1‒22.7) respectively”.

“The overall prevalence of term SGA (62.4%; 95%CI 57.1‒67.5), LBW (47.3%; 95%CI 42.0‒52.7), stunting (37.3%; 95%CI 32.3‒42.6) and preterm (10.1%; 95%CI 7.3‒13.8)” (line 286-287).

**Discussion- The overall prevalence of inadequate, adequate, and excessive GWG was 47·4%, 31·2%, and 21·4% respectively. “include this finding in chapter one of the discussion and discuss them”. “Do not repeat the results in your discussion instead discuss the findings there fore rephrase well this section by Compare, Constract and Critique your findings from the previous studies”**

Response: Correction has been done accordingly in the revised manuscript (line 391-407).

“A systematic review conducted in 25 LMICs reported that 54% and 22% of women had inadequate and excessive GWG, respectively [30]. Although the prevalence of IGWG in our study was lower than that reported in the review, the proportion with EGWG was comparable. The review highlighted nutritional, behavioural, and clinical determinants as key drivers of IGWG. Compared with another study from India [31], our cohort had a higher prevalence of both IGWG and EGWG, and a lower prevalence of AGWG. That study reported a strong association between lower energy and fat intake and IGWG, underscoring the importance of dietary quality and quantity during pregnancy.

In our setting, pregnant women generally consumed less food than the amounts recommended by the Indian Council of Medical Research, which may be related to pregnancy-related symptoms such as loss of appetite, abdominal fullness, and bloating [31]. In addition, women may have eaten smaller portions and a less diverse diet because of household dietary norms, gendered food allocation, and constrained food availability. Together, these findings suggest that both inadequate and excessive GWG in this population are shaped by a complex interplay of individual symptoms, nutritional intake, and structural barriers to food access, and highlight the need for context-specific dietary counselling and social support interventions during pregnancy.”

**Conclusion- Rephrase your conclusion to match with your results and discussion**

Response: Conclusion has been rewritten accordingly (line 436-438).

“This study demonstrates that underweight women with inadequate gestational weight gain face the highest risk of adverse outcomes compared to normal BMI and adequate gestational weight gain.

**Recommendation- Separate the conclusion and recommendation in different parts**

Response: Recommendation part has been separated (line 440-442).

“Regular monitoring of gestational weight gain is essential, and timely energy and protein supplementation should be ensured for women who are underweight and/or experience inadequate weight gain during pregnancy.”

**Limitation- Include limitation after discussing your findings**

Response: The limitations paragraph has been shifted to after discussion section (line 421-427).

“The study has some limitations. Maternal weight was not recorded at birth to calculate the total weight gain during entire pregnancy. Additionally, data on potential confounders, such as physical activity, and dietary intake were not collected, which could have provided a more comprehensive understanding of the factors influencing GWG and pregnancy outcomes. Lastly, infant anthropometric outcomes were assessed within <14 days of birth, which may have introduced some variability in measurements due to postnatal weight and length changes during this period”.

**Supporting documents- OK**

Response: Thanks

**Other section- OK**

Response: Thanks

**Change references to most recently published papers for using mostly updated reference Review all references atleast 2015 and above**

Response: All references were thoroughly reviewed and updated to include the most recent literature published from 2015 onward. Two references published prior to 2015 were retained, as they remain essential to the context and methodology of the study.

**Reference 17:** Institute of Medicine (IOM). Weight gain during pregnancy: Reexamining the guidelines. Institute of Medicine (US) and National Research Council (US) and Committee to Reexamine IOM Pregnancy Weight Guidelines 2009, was retained because gestational weight gain was categorized as inadequate, adequate, and excessive based on these guidelines, and no alternative or updated reference provides equivalent standardized criteria.

**Reference 20:** World Health Organization. WHO Child Growth Standards based on length/height, weight and age. Acta Paediatrica Supplement. 2006;450:76–85, was retained because stunting and wasting were defined as length-for-age and weight-for-length z-scores < −2 SD, respectively, according to WHO growth standards, which remain the global reference for child growth assessment.

**General comments- The work is Excellence and the idea is novel**

Response: We sincerely thank the reviewer for the positive and encouraging comments.

**Comments of Reviewer-2**

**Introduction:**

**The background and the aim that have been identified for the study is commendable and of social importance. Low birth weight has been identified as one of the causes of metabolic syndrome in that Index child and prevention of Low birth weight by identifying the reason in the Antenatal period would reduce the incidence of individuals with metabolic syndromes in a society. Stunting and Wasting at birth also have long term implications and should be prevented.**

Response: We thank the reviewer for the positive comments and for acknowledging the public health relevance of the study.

**Materials and methods**:

**The analysis used data from Women and Infants Integrated Intervention for Growth Study**

**(WINGS). 4 authors of the WINGS are involved in this study. Women recruited in the previous study (WINGS) - those with interventions and control group were followed up till they became pregnant. Those who became pregnant were recruited for this current study and randomised again to receive interventions or receive routine care.**

**The data collection method is clear. Methods of Classification of the Subjects based on BMI and follow up of the recruited subjects for Gestational weight gain has been explained adequately. The method used for the calculation of Gestational age at Birth needs more clarity. Measurements of Newborns which are needed to assess Stunting and Wasting has been mentioned clearly.**

Response: We thank the reviewer for the constructive and positive comments. We appreciate the reviewer’s acknowledgement. The calculation of gestational age at birth has now been clearly explained in the revised manuscript (line 219-222) and is addressed in response to your subsequent comment.

**Enrolment of the subjects in the current study:**

**The process of enrolment has to be worded appropriately as follows for better understanding:**

**Women from the previous study who became pregnant within 18 months of enrolment were enrolled for this study. Their BMI was calculated for classification into Women who were Underweight, Normal, Overweight and Obese. They were randomised to receive intervention or routine care.**

Response: Thank you for your suggestion. The paragraph has been rewritten (line 187-190).

“Women from the previous study [2] who became pregnant within 18 months of enrolment were included in the present study. Early pregnancy BMI was calculated and women were classified as underweight, normal weight, overweight or obese. Eligible women were then randomised to receive either the intervention or routine antenatal care”.

**Definition of primary exposure variables: Explained clearly.**

Response: Thank you very much.

**Definition of outcome variables:**

**Details of measurements of Continuous and Categorical variables have been explained well and it is found that the authors have adhered to previous prescribed standards.**

**Calculation of Exact Gestational age at birth need clarity.**

Response: Thank you for the comment. The description of the calculation of gestational age at birth has been revised and clarified (line 219-222).

“Gestational age in weeks at birth was calculated by estimating the duration in weeks from the date of the ultrasound examination to the date of birth, and adding this to the gestational age in weeks at the time of the ultrasound, as determined using the INTERGROWTH-21 standards”.

**Inclusion criteria:**

**Should be described as Antenatal women with Gestational age <14 weeks**

**Singleton pregnancy.**

**Neonates delivered by these women within <14 days of birth.**

**Further there is no comment on inclusion or exclusion of women with comorbidities which could be confounding factors.**

Response: Inclusion criteria has been corrected accordingly (line 223-225).

“The following inclusion criteria were set for this analysis: antenatal women with gestational age <14 weeks at enrolment, singleton pregnancy, and neonates delivered by these women within <14 days of birth”.

**Power Calculation and Statistical methods: Seem to be appropriate.**

Response: Thank you and we appreciate your comment.

**Table1:**

**Under this topic, the following lines may be removed, because Table 1 describes Sociodemographic profiles of Enrolled Subjects.**

**The overall prevalence of inadequate, adequate, and excessive GWG was 47·4%, 31·2%, and**

**238 21·4% respectively. Mean (SD) of newborns weight and length were 2729.9 (419.7) g and 239 48.1 (2.1) cm respectively**

Response- These lines have been shifted below table 1, and additionally, as per another reviewer’s comment, the prevalence estimates have been revised to include the corresponding 95% confidence intervals (line 271-273).

“The overall prevalence of inadequate, adequate, and excessive GWG was 47·4% (95%CI 45.8‒49.0), 31·2% (95%CI 29.8‒32.7), and 21·4% (20.1‒22.7) respectively. Mean (SD) of newborns weight and length were 2729.9 (419.7) g and 48.1 (2.1) cm respectively”

**Distribution of adverse birth outcomes by early pregnancy BMI and GWG:**

**Under this Topic, the following sentence may be rephrased or removed.**

**The overall prevalence of term SGA, LBW, preterm and stunting are shown by GWG and**

**254 early pregnancy BMI**

Response: The sentence has been removed from the manuscript.

**Association between early pregnancy BMI, GWG, and preterm:**

**Confounding factors which were considered for adjustment to be mentioned.**

Response: Thank you for suggestion. Confounding factors have been mentioned in the revised manuscript (line 317-318)

“After adjusting for maternal age, maternal education, family wealth quintile, and intervention effect, RRs for total GWG in preterm birth are shown in Fig 2C”

**Interaction between early pregnancy BMI and GWG:**

**Figures mentioned as 4A and 4B are missing**

Response: Figures 4A and 4B, along with their legends, have been cited in the manuscript (line 345-350), and figures have been provided as a separate figure file.

**“**The association between GWG (kg) per week and these outcomes was more pronounced among underweight women (BMI <18·5 kg/m²) compared to overweight or obese women (BMI ≥25 kg/m²), as illustrated in **Fig 4A and 4B** respectively. [***Fig 4A****. Relationship between GWG during pregnancy and infant weight stratified by early pregnancy BMI;* ***Fig 4B****. Relationship between GWG during pregnancy and infant length stratified by early pregnancy BMI*]”

**Discussion:**

**Appropriate.**

**Strengths and Limitations have been addressed well.**

**Among Limitations, the potential confounding factors not mentioned are Gestational diabetes, Hypertension, Anemia and other comorbidities which could have influenced Gestational weight gain and Infant birth measurements.**

Response: We thank the reviewer for this important observation. The selection of confounders has been clearly described in the Statistical Analyses section (lines 240–243).

“Confounders were included in the multivariable models based on a change-in-estimate criterion, wherein variables that altered the risk ratio (RR) or β-coefficient of the outcome variables by 5%–10% in univariable analyses were retained. Based on this predefined criterion, maternal age, maternal education, intervention status, and family wealth quintile” were identified and included as confounders in the final models. The comorbidities mentioned by the reviewer (gestational diabetes, hypertension, anemia, and other conditions) did not meet this criterion and therefore were not included in the multivariable analyses.

 “The study has some limitations. Maternal weight was not recorded at birth to calculate the total weight gain during entire pregnancy. Additionally, data on potential confounders, such as physical activity, and dietary intake were not collected, which could have provided a more comprehensive understanding of the factors influencing GWG and pregnancy outcomes. Lastly, infant anthropometric outcomes were assessed within <14 days of birth, which may have introduced some variability in measurements due to postnatal weight and length changes during this period” (page 421-427).

**Conclusion**

**Well-conceived and expressed.**

Response: Thank you very much and we appreciate your comment.

**Comments of Editor**

**Please include the following items when submitting your revised manuscript:**

- **A letter that responds to each point raised by the editor and reviewer(s). You should upload this letter as a separate file labelled 'Response to Reviewers'.**

Response- All comments from the reviewers and the editor have been addressed in the file titled ‘Response to Reviewers’

- **A marked-up copy of your manuscript that highlights changes made to the original version. You should upload this as a separgate file labelled 'Revised Manuscript with Track Changes'.**

Response- File name of changed version is ‘*Revised Manuscript with Track Changes*’

- **An unmarked version of your revised paper without tracked changes. You should upload this as a separate file labelled 'Manuscript'.**

Response- File name of cleaned version is ‘*Manuscript*’.

**Journal Requirements:**

**1. Please amend your detailed Financial Disclosure statement. This is published with the article. It must therefore be completed in full sentences and contain the exact wording you wish to be published.**

Response: Source of funding has been mentioned in the revised manuscript (line 446-447).

“This study was funded by Bill & Melinda Gates Foundation, USA (grant ID OPP1191052).”

**1. Please clarify all sources of funding (financial or material support) for your study. List the grants (with grant number) or organizations (with url) that supported your study, including funding received from your institution. 
2. State the initials, alongside each funding source, of each author to receive each grant.**

**3. State what role the funders took in the study. If the funders had no role in your study, please state: “The funders had no role in study design, data collection and analysis, decision to publish, or preparation of the manuscript.”**

Response: Thank you for the comment. Source of funding and the role of the funding agency has been clearly described in the manuscript (lines 446–449).

“This study was funded by Bill & Melinda Gates Foundation, USA (grant ID OPP1191052), awarded to NB. The funding agency had no role in designing the study, data collection, analysis, interpretation of the results, preparation of the manuscript, or decision to submit it for publication”.

**4. If any authors received a salary from any of your funders, please state which authors and which funders.**

Response: No

**2. Please provide separate figure files in .tif or .eps format.**

**For more information about figure files please see our guidelines:**

**For more information about figure files please see our guidelines:** 
<https://journals.plos.org/globalpublichealth/s/figures> 
<https://journals.plos.org/globalpublichealth/s/figures#loc-file-requirements>

Response: Figure files have been converted in .tif and uploaded as separate file

**3. Please insert an Ethics Statement at the beginning of your Methods section, under a subheading 'Ethics Statement'. It must include:**

**1) The name(s) of the Institutional Review Board(s) or Ethics Committee(s)**

**2) The approval number(s), or a statement that approval was granted by the named board(s)**

**3) (for human participants/donors) - A statement that formal consent was obtained (must state whether verbal/written) OR the reason consent was not obtained (e.g. anonymity). NOTE: If child participants, the statement must declare that formal consent was obtained from the parent/guardian.**

Response: ‘Ethics statement’ has been added as subheading under the main heading (Materials and Methods) in the manuscript (line 135-143).

“The Ethics Review Committee of the Society for Applied Studies, New Delhi (SAS/ ERC/LG/2027), Vardhman Medical College, Safdarjung Hospital, New Delhi (IEC/SJH/VMMC/PROJECT-2017/694) and the World Health Organization, Geneva (ERC.0002934) have approved the study. The study was conducted in accordance with the ethical principles outlined in the Declaration of Helsinki. The trial was registered with the Clinical Trials Registry of India (CTRI/2017/06/008908). Written informed consent was obtained from all mothers prior to participation. All data were collected in a private area of the participants’ homes, and confidentiality of the collected data was maintained”.

**4. If the reviewer comments include a recommendation to cite specific previously published works, please review and evaluate these publications to determine whether they are relevant and should be cited. There is no requirement to cite these works unless the editor has indicated otherwise.** 

Response: Reviewers did not recommend any specific published work. Only those references that were found to be directly relevant and scientifically appropriate have been cited in the manuscript.

**5. Please review your reference list to ensure that it is complete and correct. If you have cited papers that have been retracted, please include the rationale for doing so in the manuscript text, or remove these references and replace them with relevant current references. Any changes to the reference list should be mentioned in the rebuttal letter that accompanies your revised manuscript. If you need to cite a retracted article, indicate the article’s retracted status in the References list and also include a citation and full reference for the retraction notice.**

Response: Thank you for this important comment. We have carefully reviewed the entire reference list to ensure that it is complete, accurate, and up to date. All cited references were cross-checked for correctness and publication status. No retracted articles are included in the manuscript.

In accordance with one reviewer’s comment, all references were thoroughly reviewed and updated to include the most recent literature published from 2015 onward. Two references published prior to 2015 were retained, as they remain essential to the context and methodology of the study.

**Reference 17:** Institute of Medicine (IOM). Weight gain during pregnancy: Reexamining the guidelines. Institute of Medicine (US) and National Research Council (US) and Committee to Reexamine IOM Pregnancy Weight Guidelines 2009, was retained because gestational weight gain was categorized as inadequate, adequate, and excessive based on these guidelines, and no alternative or updated reference provides equivalent standardized criteria.

**Reference 20:** World Health Organization. WHO Child Growth Standards based on length/height, weight and age. Acta Paediatrica Supplement. 2006;450:76–85, was retained because stunting and wasting were defined as length-for-age and weight-for-length z-scores < −2 SD, respectively, according to WHO growth standards, which remain the global reference for child growth assessment.

**Additional Editor Comments:
As you will see from the reviews, both reviewers were enthusiastic about the manuscript but requested additional clarity in terms of methods and potential limitations. Please pay particular attention to making sure all of the possible limitations are described.**

Response: We thank the Editor for the constructive guidance. In response to the reviewers’, we have carefully revised the manuscript to enhance clarity in the methods and limitations sections to comprehensively describe all potential limitations of the study and other corrections. These revisions have been incorporated in the revised manuscript.

**Reviewers' comments:**
**Reviewer's Responses to Questions**

**Comments to the Author
1. Does this manuscript meet PLOS Global Public Health’s** [**publication criteria**](https://track.editorialmanager.com/CL0/https:%2F%2Fjournals.plos.org%2Fglobalpublichealth%2Fs%2Fcriteria-for-publication/1/010f019b213dacb5-1625614d-7d01-42c3-956a-151b5399936a-000000/G3uHOozc3spEzY7G3VHg2Tg6yPIXzL6vZtBUYnfE_mI=241)**? Is the manuscript technically sound, and do the data support the conclusions? The manuscript must describe methodologically and ethically rigorous research with conclusions that are appropriately drawn based on the data presented.**

**Reviewer #1: Yes**

**Reviewer #2: Yes**

**2. Has the statistical analysis been performed appropriately and rigorously?**

**Reviewer #1: Yes**

**Reviewer #2: I don't know**

**3. Have the authors made all data underlying the findings in their manuscript fully available (please refer to the Data Availability Statement at the start of the manuscript PDF file)?

The** [**PLOS Data policy**](https://track.editorialmanager.com/CL0/https:%2F%2Fjournals.plos.org%2Fglobalpublichealth%2Fs%2Fdata-availability/1/010f019b213dacb5-1625614d-7d01-42c3-956a-151b5399936a-000000/Hlo1HgVBl0SnsrQ5Hegyfe4owy_Nr1-y6qEZeavP-L0=241) **requires authors to make all data underlying the findings described in their manuscript fully available without restriction, with rare exception. The data should be provided as part of the manuscript or its supporting information, or deposited to a public repository. For example, in addition to summary statistics, the data points behind means, medians and variance measures should be available. If there are restrictions on publicly sharing data—e.g. participant privacy or use of data from a third party—those must be specified.**

**Reviewer #1: Yes**

**Reviewer #2: No**

**4. Is the manuscript presented in an intelligible fashion and written in standard English?

PLOS Global Public Health does not copyedit accepted manuscripts, so the language in submitted articles must be clear, correct, and unambiguous. Any typographical or grammatical errors should be corrected at revision, so please note any specific errors here.**

**Reviewer #1: Yes**

**Reviewer #2: No**

**5. Review Comments to the Author**
**Please use the space provided to explain your answers to the questions above. You may also include additional comments for the author, including concerns about dual publication, research ethics, or publication ethics. (Please upload your review as an attachment if it exceeds 20,000 characters)**

**Reviewer #1: The work is Excellence and the idea is novel. However, the authors needs to address these minor comments. The authors need to review the abstract, methodology, ethical issues, discussion, conclusion and recommendation and references but add the limitation section which is not included in the manuscript.**

Response: We sincerely thank the reviewer for the positive appraisal of our work and for acknowledging the novelty of the idea. In response to the reviewer’s comments, we have carefully reviewed and revised the abstract, methodology, ethical considerations, discussion, conclusion, recommendations, and references to improve clarity and completeness. In addition, a **limitation** section has been included in the manuscript and positioned after the discussion section, where all potential limitations of the study are clearly described in the revised.

**Reviewer #2: This Study seems to be an extension of Previous published Study -WINGS. Data from that Study has been used and it has been declared by the Authors. Statistical Analyses has to be checked by Domain experts. Certain Tables and Figures mentioned in the text are not available for perusal. Few corrections in presentation and wordings are mentioned in my report which would be uploaded.**

Response: Thank you for the reviewer’s careful evaluation and constructive suggestions. In response to the comment regarding statistical analyses, all analyses have been rechecked and necessary clarifications and corrections have been incorporated in the methods and Results sections.

Regarding the unavailability of certain tables and figures mentioned in the text, we confirm that all referenced tables and figures have now been properly included, correctly numbered, and made available for review in the revised manuscript. Additionally, all suggested corrections related to presentation and wording, as indicated in the reviewer’s detailed report, have been carefully addressed in the revised manuscript.
