# Supplementary material for: Impact of early pregnancy body mass index and gestational weight gain on birth outcomes: Findings from a pregnancy cohort in South Delhi, India
Source: PLOS Glob Public Health. 2026 Feb 9;6(2):e0005932. doi: 10.1371/journal.pgph.0005932 (PMC12885321; doi:10.1371/journal.pgph.0005932)
Supplement: Attachment — Submitted filename: CORRECTED REVIEW.docx [file pgph.0005932.s010.docx]

**Peer Review for PLOS Global Public Health:**

By Dr.Chandrakala Maran

HOD, OBGYN Dept, K.G.Hospital And PG Institute, Coimbatore,INDIA

**Article:**

Impact of early pregnancy body mass index and gestational weight gain on birth

outcomes: Findings from a pregnancy cohort in South Delhi, India

**Table1:**

Under this topic, the following lines may be removed, because Table 1 describes Sociodemographic profiles of Enrolled Subjects.

The overall prevalence of inadequate, adequate, and excessive GWG was 47·4%, 31·2%, and

238 21·4% respectively. Mean (SD) of newborns weight and length were 2729.9 (419.7) g and 239 48.1 (2.1) cm respectively

**Interaction between early pregnancy BMI and GWG:**

Figures mentioned as 4A and 4B are to be checked. May have been submitted separately.If found to be appropriate,It may be accepted.

**Discussion:**

Among Limitations, the potential confounding factors not mentioned are Gestational diabetes,Hypertension ,Anemia and other comorbidities which could have influenced Gestational weight gain and Infant birth measurements.

Please mention if Women with the above mentioned medical disorders were excluded.

The Article may be accepted after making these minor changes.
