# Supplementary material for: Impact of early pregnancy body mass index and gestational weight gain on birth outcomes: Findings from a pregnancy cohort in South Delhi, India
Source: PLOS Glob Public Health. 2026 Feb 9;6(2):e0005932. doi: 10.1371/journal.pgph.0005932 (PMC12885321; doi:10.1371/journal.pgph.0005932)
Supplement: Attachment — Submitted filename: Response to Reviewer_14.01.2026.docx [file pgph.0005932.s011.docx]

**Peer Review for PLOS Global Public Health:**

By Dr.Chandrakala Maran

HOD, OBGYN Dept, K.G.Hospital And PG Institute, Coimbatore,INDIA

**Article:**

Impact of early pregnancy body mass index and gestational weight gain on birth

outcomes: Findings from a pregnancy cohort in South Delhi, India

**Table1:**

**Under this topic, the following lines may be removed, because Table 1 describes Sociodemographic profiles of Enrolled Subjects.**

**The overall prevalence of inadequate, adequate, and excessive GWG was 47·4%, 31·2%, and 238 21·4% respectively. Mean (SD) of newborns weight and length were 2729.9 (419.7) g and 239 48.1 (2.1) cm respectively**

Response: Thank you for the suggestion. The paragraph has been removed from the text.

**Interaction between early pregnancy BMI and GWG:**

**Figures mentioned as 4A and 4B are to be checked. May have been submitted separately. If found to be appropriate, it may be accepted.**

Response: Thank you for your comments. Figure 4 (panels 4A and 4B) has been submitted as a separately file name “Fig 4.tif” and has been cited at the appropriate places in the text (lines 341–342).

**Discussion:**

**Among Limitations, the potential confounding factors not mentioned are Gestational diabetes, Hypertension, Anemia and other comorbidities which could have influenced Gestational weight gain and Infant birth measurements.**

**Please mention if Women with the above mentioned medical disorders were excluded.**

**The Article may be accepted after making these minor changes.**

Response: **Thank you for the suggestion.** We included gestational diabetes, hypertension, and anemia as the potential confounders in the multivariable models, however, none of the factors did not change risk ratio (RR) or β-coefficient of the outcome variables by 5% from the univariable models. We have now modified the limitations as per the reviewer suggestions (line 418-431).

“The study has some limitations. Maternal weight was not recorded at birth to calculate the total weight gain during entire pregnancy. Several potential confounders were not measured, such as physical activity, and dietary intake which could have provided a more comprehensive understanding of factors influencing GWG and pregnancy outcomes. Additionally, while we screened for clinical intermediaries such as gestational diabetes, pregnancy-induced hypertension, and anemia, these were excluded from the final multivariable models because their inclusion did not alter the risk ratios or $\beta$-coefficients of the primary outcomes by the pre-specified threshold of 5%–10%. While this parsimonious approach focuses on the most statistically influential predictors, we acknowledge that these clinical conditions remain biologically relevant. Their exclusion might limit our ability to explore specific pathological pathways, though it ensures the stability and interpretability of our primary associations. Lastly, infant anthropometric outcomes were assessed within <14 days of birth, which may have introduced some variability in measurements due to postnatal weight and length changes during this period.”
