# Supplementary material for: Impact of early pregnancy body mass index and gestational weight gain on birth outcomes: Findings from a pregnancy cohort in South Delhi, India
Source: PLOS Glob Public Health. 2026 Feb 9;6(2):e0005932. doi: 10.1371/journal.pgph.0005932 (PMC12885321; doi:10.1371/journal.pgph.0005932)
Supplement: Attachment — Submitted filename: Reviewer COMMENTS for Manuscript ID PGPH-D-25-02926.pdf [file pgph.0005932.s007.pdf]

REVIEWER'S COMMENTS

PLOS Global Public Health

Impact of early pregnancy body mass index and gestational weight gain on birth outcomes: Findings from a pregnancy cohort in South Delhi, India

Manuscript ID : PGPH-D-25-02926

S/N	Section	Comments from the reviewer	PI's response	Line number
1	**Title**	OK		
2	**Abbreviations**	Give the list of all abreviations with their long term		NA
3	**Abstract**	Write the name of corresponding author and email		14
		Write the section of abstract into strcutured paragraphs		26-53
		The highest prevalence of SGA (62.4%) was observed among underweight women 40 with IGWG.<br>Give the CI for this prevalance value don't leave it alone		39-40
4.	**Introduction**	Ok	NA	NA
5.	**Research questions or hypothesis**	OK	NA	NA

4.	**Methodology**	Prepregnant women were enrolled<br>117 from 1 July 2017 to 30 December 2019 and were prospectively followed, and birth outcomes<br>118 were collected upto 10 July 2021 " State the follow up time by saying were followed for the period of …. Years from ….. to ….."	NA	117-118
	**Inclusion criteria:**	The following inclusion c 196 riteria were set for this analysis: <14 weeks of<br>197 gestational age at enrolment, singleton pregnancy and birth outcomes were measured <14<br>198 days of birth.	OK	NA
	**Exclusion creteria**	Include the exclusion creteria in your work What were the exclusion creterias		
	**Analysis**	All analyses were performed using Stata version 14 (StataCorp,		226-227

		College Station, TX, USA),<br>227 with statistical significance defined as p < 0.05. " Include the words from 95% CI at the end of this sentence"		
5.	**Ethical Issues**	Show how the confidentiality, Privacy and conserting was done as well as how participants were enrolled ethically	NA	159-163
6.	**Results**	The overall prevalence of inadequate, adequate, and excessive GWG was 47·4%, 31·2%, and<br>238 21·4% respectively.<br>" Put the CI in this prevalance"	NA	237-238
7.	**Discussion**	The overall prevalence of inadequate, adequate, and excessive GWG was 47·4%, 31·2%, and<br>238 21·4% respectively.<br>" include this findings in chapter one of the discusion and dicsuss them"	NA	319-391

		"Do not repeat the results in your dicussion in stead discuss the findings there fore rephrse well this section by Compare, Constract and Critique your findings from the previous studies"		
	**Conclusion**	Reprhase your conclusion to match with your results and dicsussion		394-398
	**Recommendation**	Sepaate the conclusion and recommendation in different parts		398-406
	**Limitations**	Include limitation after discussing your findings		
8.	**Supporting documents**	OK	NA	NA
9.	**Other section**	Ok	NA	NA
10.	**References**	Change references to most recently publushed papers for using mostly updated reference Review all references atleat 2015 and above		References section
11.	**General Comments**	The work is Excellence and the idea is novel		NA

		However, the authors needs to adress these minor comments		
